# Therapeutic effects of umbilical cord blood plasma in a rat model of acute ischemic stroke

**DOI:** 10.18632/oncotarget.12998

**Published:** 2016-10-31

**Authors:** Jongman Yoo, Han-Soo Kim, Jin-Ju Seo, Jang-Hyoun Eom, Seong-Mi Choi, Sanghyun Park, Dong-Wook Kim, Dong-Youn Hwang

**Affiliations:** ^1^ Department of Microbiology and Institute of Basic Medical Sciences, School of Medicine, CHA University, Seongnam, Kyeonggido 463-400, Korea; ^2^ Department of Biomedical Sciences, Catholic Kwandong University, Gangneungsi, Gangwondo 210-701, Korea; ^3^ Department of Biomedical Science, College of Life Science, CHA University, Seongnam, Kyeonggido 463-400, Korea; ^4^ Department of Physiology and Brain Korea 21 Plus Project for Medical Science, Yonsei University College of Medicine, Seoul 120-752, Korea

**Keywords:** stroke, umbilical cord plasma, ischemia, neuroinflammation, neurogenesis

## Abstract

Umbilical cord blood plasma (UCB-PL) contains various cytokines, growth factors, and immune modulatory factors that regulate the proliferation and function of immune cells and adult stem cells. Despite its therapeutic potential, the effects of UCB-PL treatment in conditions of ischemic brain injury have yet to be investigated. In this study, we demonstrated that both behavioral and structural impairments resulting from ischemic brain injury were significantly prevented/reversed after intravenous administration of UCB-PL relative to the vehicle control. As early as 1-week post-ischemia, an increased number of newborn cells in the subventricular zone and a reduced number of activated microglial cells in the peri-infarct area were observed in the UCB-PL group, suggesting that enhanced neurogenesis and/or the suppression of inflammation may have contributed to functional protection/recovery. Moreover, UCB-PL was more effective than plasma derived from a 65-year-old healthy adult for the treatment of ischemia-related structural and functional deficits, indicating that UCB-PL had greater therapeutic potential. This study provides valuable insights into the development of a safe, effective, and cell-free strategy for the treatment of ischemic brain damage and a much-needed alternative for patients who are ineligible for thrombolytic therapy.

## INTRODUCTION

Stroke, a devastating cerebrovascular disease, is classified into ischemic and hemorrhagic subtypes based on its etiology [1, 2]. More than three-quarters of all cases are reported to result from ischemic injury. Thrombolytic therapy is the first line therapy for ischemic stroke. However, the window of effectiveness of tissue plasminogen activator (tPA) treatment is only 4.5 h post-ischemic injury, which allows only 8% of stroke patients to receive tPA therapy [3, 4]. New cell-based therapeutic strategies are in development that use bone marrow- or adipose tissue-derived mesenchymal stem cells (MSCs) and neural stem cells. However, in the case of stem cell administration to patients, the potential risk of serious side effects such as uncontrolled growth of the transplanted cells [5] and clogging of small blood vessels by occasional unexpected cell aggregates cannot be avoided. Therefore, from a clinical safety perspective, cell-free therapeutic strategies are more desirable if comparable efficacy can be achieved. Although conditioned media of MSCs were previously tested as cell-free therapeutic strategies [6, 7], the exposure to fetal bovine serum during the initial establishment and expansion of the MSCs raised concerns of xenopathogen transmission.

Umbilical cord blood contains a large number of stem cells (i.e., MSCs and hematopoietic stem cells). Human umbilical cord blood plasma (UCB-PL) contains a variety of cytokines, growth factors, and immune modulatory factors that affect the proliferation and function of immune cells and MSCs [8–11]. Given the beneficial constituents of UCB-PL, it may have therapeutic utility for the treatment of a variety of disease states. Furthermore, no risk of xenopathogen transmission is associated with therapies that use UCB-PL.

In this study, we demonstrate that UCB-PL administration exerted therapeutic effects on both structural and behavioral recovery in a rat model of focal ischemic stroke. The results of this study provide valuable insights into the development of a safe, alternative, and cell-free therapeutic strategy for stroke patients who are ineligible for thrombolytic therapy.

## RESULTS

### UCB-PL administration promotes behavioral recovery in a rat model of acute ischemic stroke

**Figure 1:**
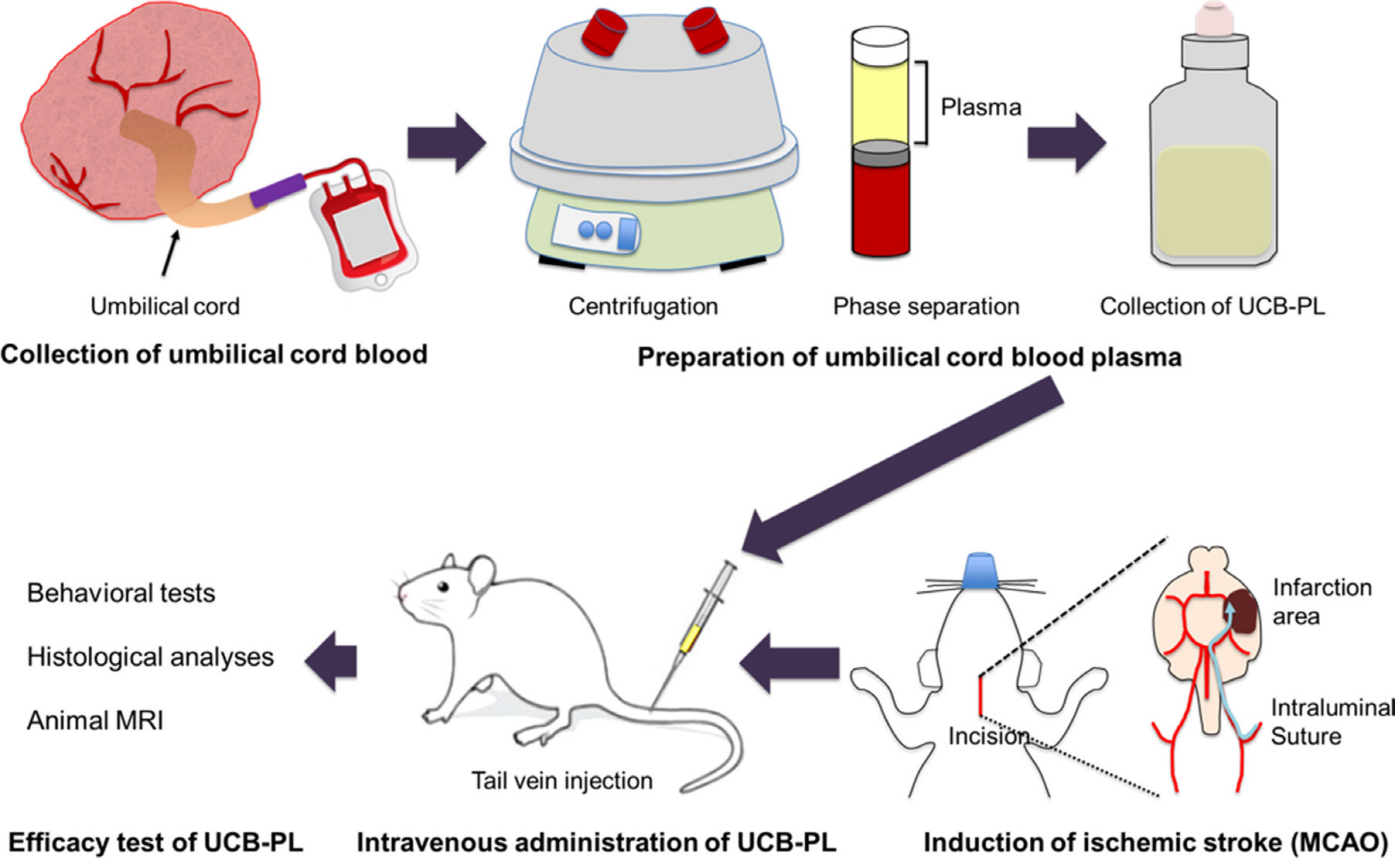
The experimental study design

**Figure 2:**
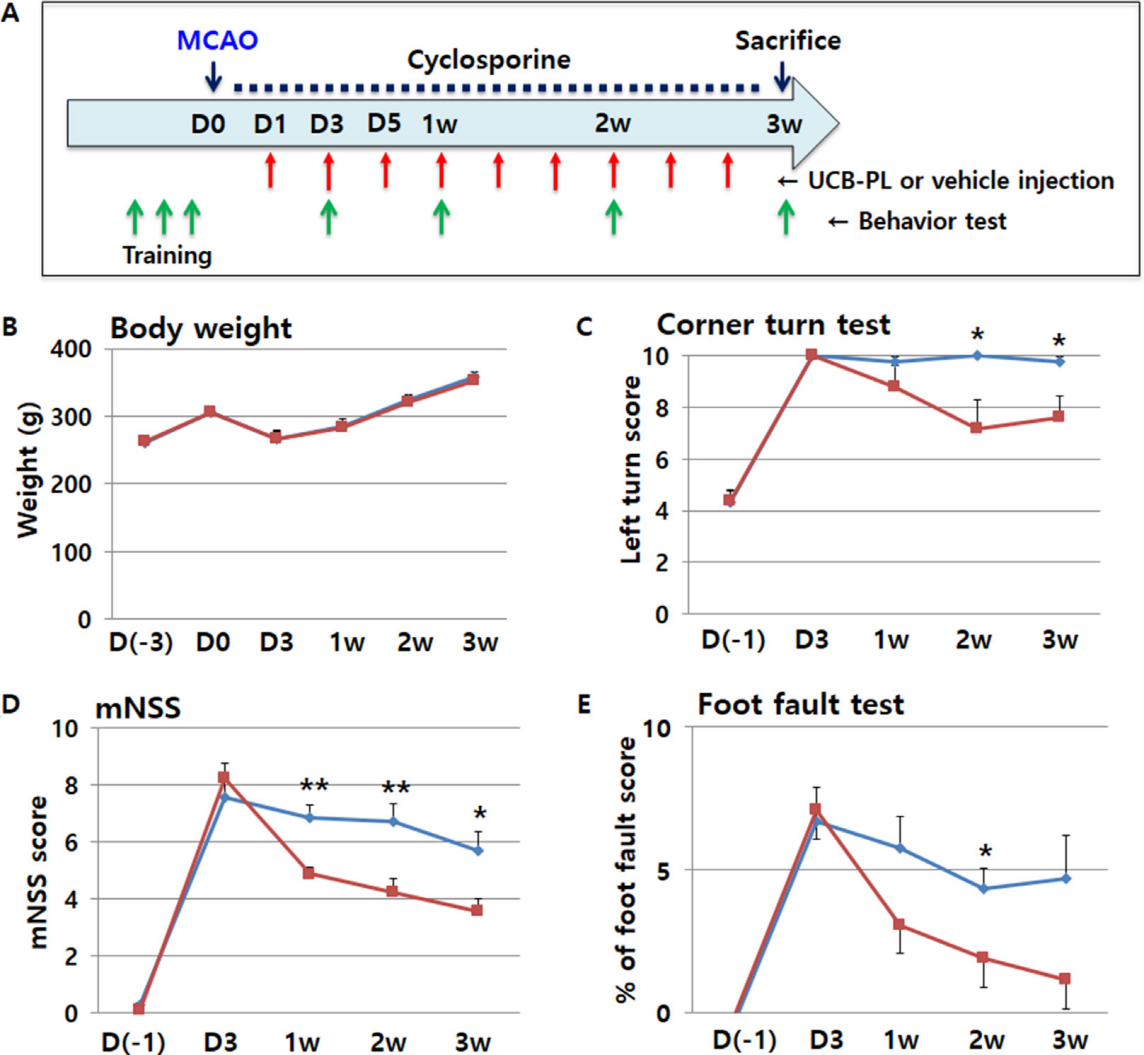
Functional recovery after UCB-PL administration in an acute ischemic stroke model UCB-PL was intravenously administered during the acute phase of a rat model of stroke, and effects on behavioral performance were measured. (**A**) The schedule of the experiment. (**B**) The physiological status and health of the rats in each group were measured using body weight. (**C**–**E**) Behavioral performances of rats in each group were measured using the corner turn test (C), the modified neurologic severity score (mNSS) (D), and the foot fault test (E). *n* = 7 for the control group (blue line), and *n* = 9 for the UCB-PL group (red line). **p < 0.01, *p < 0.05; data are presented as the mean ± SEM.

The experimental design is illustrated in Figure 1. Behavioral performances were examined between the control (PBS-treated) and UCB-PL groups, which had received treatment every 2 days starting from the third day after MCAO surgery for a total of 9 treatments (Figure 2A). Behavioral assessments were performed on days 3, 7, 14, and 21 post-MCAO surgery, and animals were euthanized after the last behavioral test on day 21 for histological analysis (Figure 2A).

No significant differences in body weight were observed between the control group and the UCB-PL group (Figure 2B). The UCB-PL group displayed significant improvements in the corner turn test compared with the control group from the first week after MCAO surgery; this functional difference became statistically significant from the second week after MCAO surgery (Figure 2C). The UCB-PL group also showed significant differences in neurological recovery as indicated by the mNSS as early as 1 week after MCAO surgery (Figure 2D). Lastly, the UCB-PL group showed better performance in the foot fault test compared with the control group (Figure 2E). Taken together, these behavioral analyses suggest that intravenous UCB-PL attenuated or reversed behavioral impairments after MCAO.

### UCB-PL administration reduces brain tissue damage after MCAO

**Figure 3:**
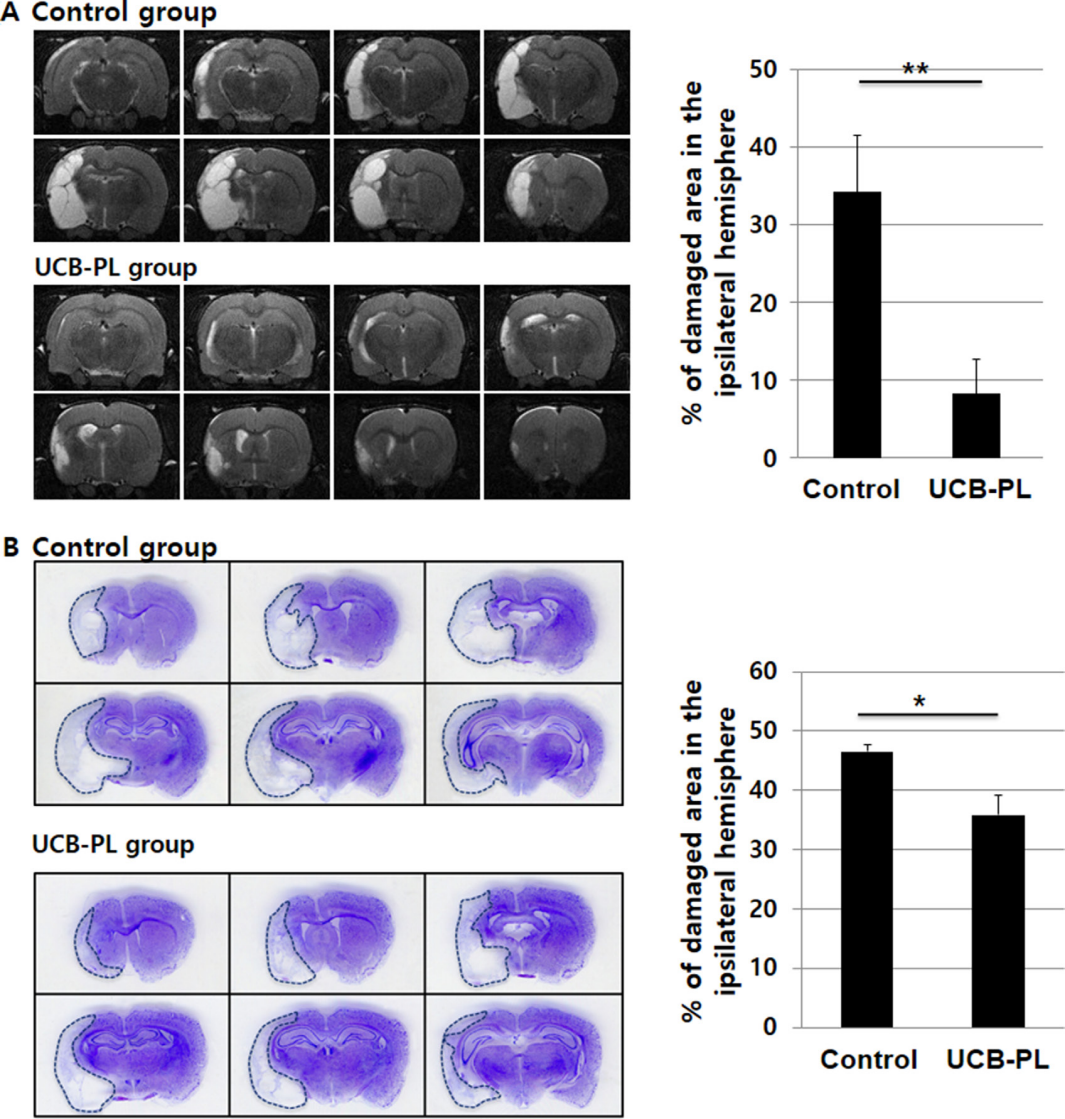
UCB-PL administration reduced brain tissue damage caused by MCAO injury (**A**) (*Left panel*) *In vivo* brain MRI was performed prior to euthanasia to measure the size of the damaged brain area. (*Right panel*) The percentage of the damaged area in the ipsilateral hemisphere was compared between the control and UCB-PL groups. Eight sections per rat were analyzed to quantify the percentage of the remaining intact ipsilateral area. (**B**) (*Left panel*) Cresyl violet staining of the rat brains was performed to measure the size of the damaged brain area. (*Right panel*) The graph indicates the percentages of the hemispheres that were damaged by ischemia in each group. *n* = 6/group, *p < 0.05, **p < 0.01.

Because behavioral improvements were evident following UCB-PL administration, we examined whether UCB-PL also limited the extent of or enhanced recovery from MCAO-mediated brain damage. MCAO-associated brain damage was measured using *in vivo* MRI prior to euthanasia. The UCB-PL group displayed a significant reduction in brain tissue damage compared to the control (PBS) group (8.3% in the UCB-PL group [*n* = 6] vs. 34.2% in the control group [*n* = 6], *p* < 0.05) (Figure 3A).

Histological analysis after sacrificing the rats also showed significant brain tissue recovery in the UCB-PL group compared to the control group. The reduced brain volumes were 47% and 36% of the hemisphere in the control and UBC-PL groups, respectively (*n* = 6, *p* < 0.05) (Figure 3B).

This result suggested that UCB-PL treatment was effective for the prevention or reversal of brain damage resulting from ischemic injury, which is consistent with our behavioral observations.

### UCB-PL administration enhances neural stem cell proliferation in the subventricular zone after MCAO

**Figure 4:**
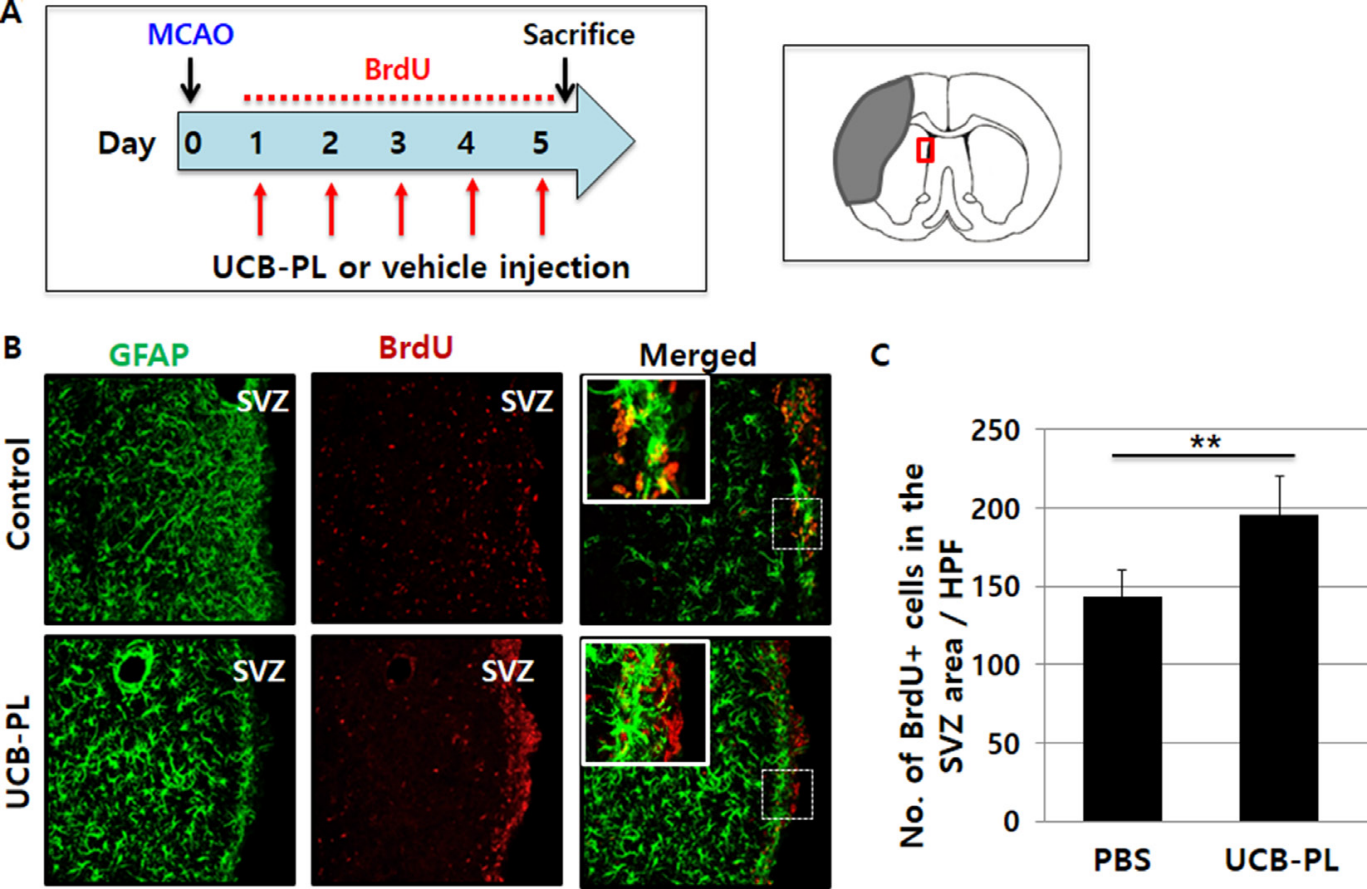
Increased neural stem cell proliferation in the subventricular zone (SVZ) after UCB-PL administration (**A**) The schedule of the experiment; BrdU, 5-bromo-2-deoxyuridine. (**B**) The expression levels of GFAP and BrdU in the SVZ were compared between the control and UCB-PL groups. (**C**) The number of BrdU-positive cells per high-power field was counted. *n* = 5/group, **p < 0.01, scale bar: 10 μm.

To explore the mechanism underlying the functional and structural recovery elicited by UCB-PL treatment, we first examined whether UCB-PL administration increased the proliferation of otherwise quiescent neural stem cells in the subventricular zone (SVZ). Rats were injected with BrdU for 5 consecutive days starting 1 day after MCAO surgery and were euthanized 1 day after the last BrdU injection (Figure 4A). The number of BrdU^+^ cells in the SVZ of the infarcted hemisphere was significantly greater in the UCB-PL group than in the control group (1.36-fold, *n* = 5, *p* < 0.01) (Figure 4B, 4C). Our results demonstrated that UCB-PL administration during the acute stage of stroke enhanced the proliferation of neural stem cells in the SVZ, suggesting that this effect at least in part accounted for the observed behavioral and structural recovery after ischemic brain injury.

### UCB-PL administration decreases microglial activation after MCAO

**Figure 5:**
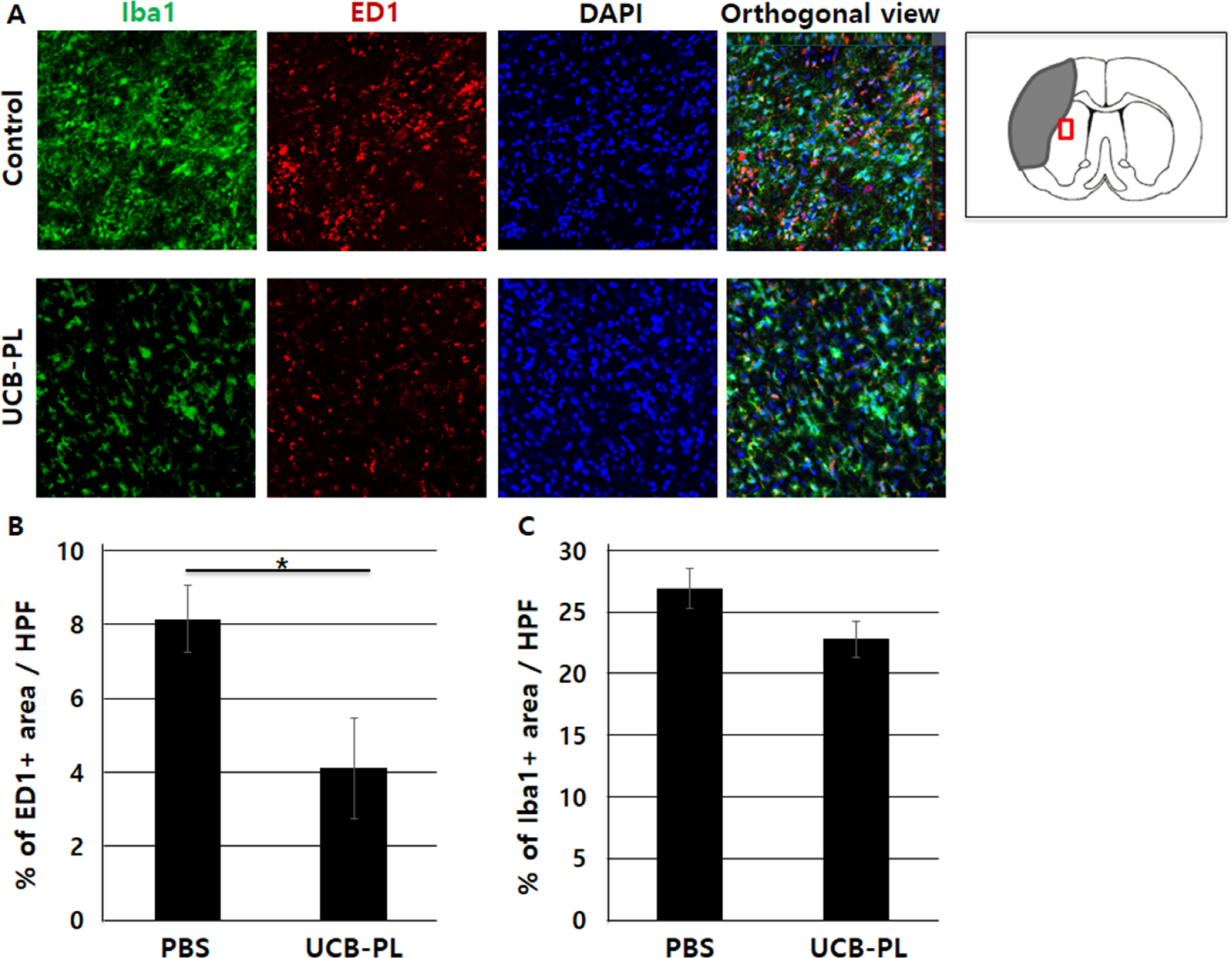
Reduced microglial activation after UCB-PL administration (**A**) The expression levels of Iba1 and ED1 in the striatum were compared between the control and UCB-PL groups. (**B**) The percentage of ED1-positive area per high-power field was measured. (**C**) The percentage of Iba1-positive area per high-power field was measured. *n* = 4/group, *p < 0.05, scale bar: 10 μm.

It has been suggested that limiting the extent of microglial activation after injury promotes neural proliferation and reduces post-ischemic brain damage [12–14]. When rat brain slices were immunostained for both Iba1, a general marker for microglia, and ED1, a marker specific for activated microglia, an approximately 50% reduction in ED1^+^ area was observed near the peri-infarct striatum in the UCB-PL group relative to the control group (0.5-fold, *n* = 4, *p* < 0.05) (Figure 5A, 5B). However, we also observed a slight reduction in Iba1^+^ area near the peri-infarct striatum in the UCB-PL group relative to the control group (Figure 5C). These results indicated that UCB-PL administration significantly decreased microglial activation near the peri-infarct striatum.

### UCB-PL is more effective than aged-PL for the treatment of ischemic injury

**Figure 6:**
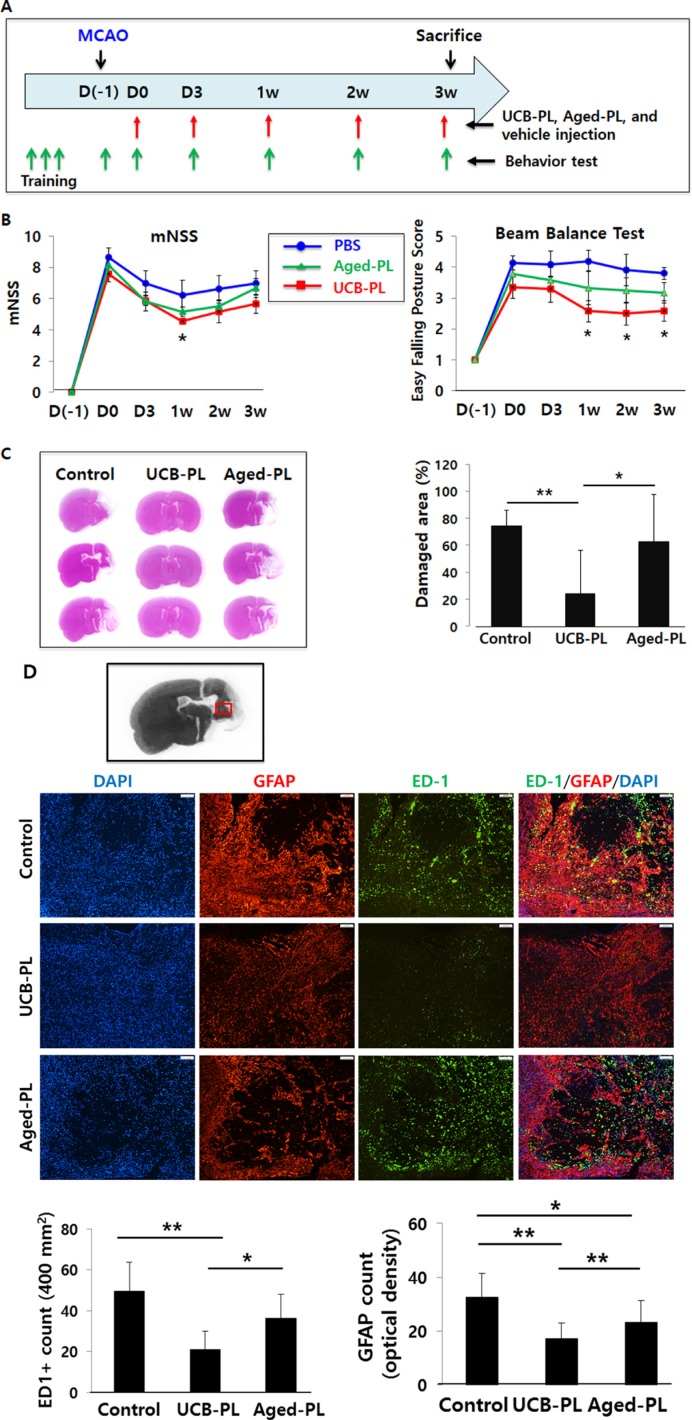
Comparison of therapeutic effects among the UCB-PL, aged-PL, and control groups (**A**) Schedule of the experiment. (**B**) The behavioral performances of rats in each group were measured with using the modified neurologic severity score (mNSS) (*left*) and the beam balance test (*right*). (**C**) The percentage of damaged area per hemisphere was measured 3 weeks after the MCAO surgery. (**D**) The expression levels of ED1 and GFAP were measured in the peri-infarct striatum and compared among the 3 groups. The number of ED1-positive cells (*right andupper graphs*) and GFAP-positive optical density (*right and lower graphs*) were measured in the peri-infarct area. *n* = 5 for the control group, *n* = 6 for the aged-PL group, and *n* = 6 for the UCB-PL group. **p < 0.01, *p < 0.05; data are presented as the mean ± SEM.

A previous study of mouse parabiosis suggested that systemic administration of young mouse plasma elicited greater improvements in cognitive function than aged mouse plasma [15]. Based on this result, we speculated that UCB-PL would offer greater benefits than aged-PL after MCAO in rats. To test this hypothesis, we administered (via the tail vein) either UCB-PL or plasma from a 65-year-old healthy subject (aged-PL) 5 times post-MCAO on days 0, 3, 7, 14, and 21, and performed both behavioral and histological analyses (Figure 6A).

With respect to the mNSS, the UCB-PL group displayed better recovery than both the aged-PL and control groups. The difference was statistically significant at 1 week after MCAO surgery (Figure 6B, *left*). In the beam balance test, which is a sensitive motor assay, the UCB-PL group showed more significant improvements in behavioral performance than the aged-PL or control groups from 1 week post-MCAO and thereafter (Figure 6B, *right*).

Nissl staining of brain sections harvested at 3 weeks post-MCAO demonstrated that the UCB-PL group displayed a lesser degree of brain damage than the aged-PL group (24.0% in the UCB-PL group [*n* = 7] vs. 63.0% in the aged-PL group [*n* = 7], *p* < 0.05) (Figure 6C).

When rat brain sections were immunostained for ED1, a marker for activated microglia, and GFAP, a marker for activated astrocytes, a 44.5% reduction in the number of ED1+ cells (per 400 mm^2^) and a 26% reduction in GFAP+ area (as measured by optical density) were noted near the peri-infarct striatum in the UCB-PL group compared with the aged-PL group (Figure 6D).

Taken together, our results indicate that UCB-PL administration in the acute phase of an ischemic stroke model prevented or reversed behavioral and functional impairments more efficiently than aged-PL or PBS administration. Therefore, UCB-PL is a novel and promising cell-free therapeutic strategy for the safe allogeneic treatment of ischemic brain injury.

## DISCUSSION

In this study, we successfully demonstrated that intravenous administration of UCB-PL improved behavioral performance post-MCAO in 3 behavioral tests, including the mNSS, the corner turn test, and the foot fault test. In support of the observed functional improvements, brain infarct size as measured by both MRI and Nissl staining was significantly reduced in the UCB-PL group. Therefore, our results indicate that intravenous administration of UCB-PL in the acute phase after MCAO in rats improved behavioral performance and reduced structural damage.

UCB-PL is known to contain a variety of soluble factors, including growth factors, cytokines, and immune modulatory factors [16–20], that have the potential to ameliorate symptoms caused by ischemic stroke. UCB-PL, which does not contain any cells, has several advantages as a new therapeutic option; first, there is no risk of tumor formation or cell aggregation. In addition, UCB-PL is convenient to obtain, store, handle, and use (i.e., it can be frozen, thawed, transported, and administered to patients with ease) for clinical application. Furthermore, unlike bone marrow or peripheral blood, the absence of immune cells in UCB-PL signifies a decreased possibility of graft vs. host disease (GvHD) or immune rejection.

At least possible 3 mechanisms for the efficacy of UCB-PL administration are supported by this study; (1) UCB-PL administration reduced neuroinflammation, as demonstrated by a reduced number of activated microglia in the peri-infarct area following UCB-PL administration; (2) UCB-PL administration increased neurogenesis in the SVZ area and, most likely, the migration of new cells to the injured area; and (3) soluble factors in UCB-PL had a paracrine effect on the injury site and caused the observed beneficial effects. Previous studies have reported that inflammation confers detrimental effects on neural proliferation in the injured brain [21, 22]. Therefore, increased neurogenesis by UCB-PL administration may be indirectly supported through the suppression of microglial activation and/or directly supported by the induction of neurogenesis by soluble factors in UCB-PL.

Intriguingly, plasma from a 65-year-old healthy subject did not produce the same beneficial effects as UCB-PL, suggesting that the composition of soluble factors in plasma from UCB and aged individuals are not only different but also have different degrees of therapeutic utility. Although beyond the scope of this study, it would be of interest to specifically identify the component(s) of UCB-PL that are responsible for the observed therapeutic effects (for example, by comparing the differences in the composition of the 2 different plasmas).

## MATERIALS AND METHODS

### Animals

This section of the study was approved by the CHA University Institutional Animal Care and Use Committee. Middle cerebral artery occlusion (MCAO) was used to generate a rat model of ischemic stroke [23]. The surgery was conducted on adult male Sprague-Dawley rats (*n* = 30 per group) (Orient Bio, Seongnam, Korea) weighing 240–270 g each. Briefly, rats were anesthetized with 5% isoflurane (Hana Pharm, Hwasung, Korea) and were maintained under anesthesia with 2% isoflurane in a mixture of 70% N_2_ and 30% O_2_. The common carotid artery, internal carotid artery (ICA), and external carotid artery (ECA) were exposed by a cervical incision, and the pterygo-palatine and occipital arteries were cauterized. Blood flow was temporarily occluded by ligating the ICA and ECA. A 23-mm-long 4–0 nylon monofilament (Ailee, Busan, Korea) coated with low-viscosity silicone (Handae Chemicals, Chungbuk, Korea) was advanced from the exposed ECA to the ICA to occlude the middle cerebral artery. Reperfusion was induced by withdrawing the suture from the ECA at 1.5 h post-ischemia.

### Preparation and administration of UCB-PL

This section of the study was approved by the Institutional Review Board of CHA Bundang Medical Center, Korea. Human UCB containing citrate phosphate dextrose adenine as an anticoagulant was obtained from the Cord Blood Bank, CHA Bundang Medical Center. Only fresh UCB supplied within 24 h postpartum was used in this study. For preparation, UCB was centrifuged for 10 min at 1500 × *g* at room temperature and separated into 3 layers; plasma (UCB-PL; top), a mixture of leucocytes and platelets (middle), and erythrocytes (bottom). The UCB-PL layer was carefully collected, aliquoted, and stored at −80°C until use. For comparison, plasma was also prepared from a 65-year-old healthy subject. The method of plasma preparation was the same as that described above. For administration, UCB-PL or control plasma (1 mL/kg) was intravenously injected slowly (1 to 2 min) into the tail vein according to the experimental schedules specified below.

### Behavioral tests

The rats that showed similar behavioral defects were assigned randomly to each group. For behavioral assessments, each rat was subjected to a battery of behavioral tests and evaluated by an experimenter who was blinded to the treatment conditions. The modified neurologic severity score (mNSS), foot fault test, and beam balance test were performed on days −3, 0, 1, 3, 5, 7, 14, and 21 post-MCAO surgery. The mNSS was used to assess motor (muscle status and abnormal movement), sensory (visual, tactile, and proprioceptive), and reflex (pinna, corneal, and startle) functions [24]. The test results were indexed as a grade that ranged from 0 to 18 (0, no impairment; 1–6, mild impairment; 7–12, moderate impairment; and 13–18, severe impairment).

The foot fault test was performed by placing rats on a wire grate (50 cm × 50 cm with 2.5 cm × 2.5 cm grids) for 5 minutes, and the number of times the forelimbs slipped through the grate during a 5-minute session was recorded (by videotaping from below the grate) [25]. Raw numbers were converted to foot fault scores as follows:

Foot fault score = (number of faults for the affected forelimb/number of footsteps of the affected forelimb) − (number of faults for the unaffected forelimb/number of footsteps of the unaffected forelimb).

The beam balance test was performed by placing rats at the center of a beam (100 cm × 5 cm × 2 cm) and grading motor performance on a 6-point scale, as follows [26]. 1, balances with steady posture and paws on top of the beam; 2, grasps the sides of the beam and has shaky movement; 3, one or more paw slips off of the beam; 4, attempts to balance on the beam but falls off; 5, drapes over the beam but falls off; and 6, falls off of the beam without attempting to balance.

### 5-Bromo-2-deoxyuridine (BrdU) labeling

BrdU (50 mg/kg in saline) (Sigma-Aldrich, St. Louis, MO, USA) was administered intraperitoneally (i.p.) daily for 5 days starting the day after MCAO surgery. Rats were euthanized with a mixture of ketamine (50 mg/kg, i.p.) (Huons, Seoul, Korea) and xylazine (5 mg/kg, i.p.) (Bayer Korea, Seoul, Korea) and transcardially perfused with 0.1 M PBS followed by 4% formaldehyde (Samchun Chemicals, Pyeongtaek, Korea). Brains were removed and post-fixed in 4% formaldehyde overnight at 4°C and then sequentially cryoprotected in 15% and 30% sucrose (Sigma-Aldrich) in phosphate-buffered saline. Brains were then stored at −80°C in an optimal cutting temperature (OCT) solution (Sakura Finetek, Torrance, CA, USA) and finally sectioned at a thickness of 30 μm using a cryostat (Microm, HM 525, Walldorf, Germany). To perform BrdU staining, brain sections were incubated in 2 N HCl (Samchun Chemicals) at 37°C for 30 min and subsequently neutralized in 0.1 M sodium borate (pH 8) (Sigma-Aldrich) for 20 min. Sections were then blocked with 10% goat serum (Vector Laboratories, Burlingame, CA, USA) and 0.1% NP-40 (Sigma-Aldrich) in Tris-buffered saline (TBS) (Sigma-Aldrich) for 1 h, followed by incubation with 2 μg/mL of a rat monoclonal anti-BrdU antibody (Abcam) at 4°C for 12 h, and then with 2 μg/mL of a goat Alexa 594-conjugated anti-rat IgG antibody (Invitrogen) for 40 min at room temperature. Sections were finally mounted on glass slides (Marienfeld, Lauda-Koenig-Shofen, Germany) and examined with an LSM510 or LSM610 confocal microscope (Carl Zeiss, Göttingen, Germany).

### Immunohistochemistry

Immunostaining was performed as described above. The following primary antibodies were used: rabbit polyclonal anti-doublecortin (anti-DCX, 2 μg/mL) (Cell Signaling Technology, Danvers, MA, USA), mouse anti-glial fibrillary acid protein (anti-GFAP, 2 μg/mL) (Millipore), mouse anti-neuronal nuclei (NeuN, 2 μg/mL) (Millipore), rabbit polyclonal anti-ionized calcium-binding adapter molecule 1 (anti-Iba1, 2 μg/mL) (Wako Pure Chemical Industries, Osaka, Japan), and mouse monoclonal anti-macrophages/monocytes clone ED1 (anti-ED1/CD68, 2 μg/mL) (Millipore). The following secondary antibodies (4 μg/mL) were subsequently applied: goat Alexa 594-conjugated anti-rat IgG, goat Alexa 594-conjugated anti-mouse IgG, goat Alexa 594-conjugated anti-mouse IgM, and goat Alexa 594-conjugated anti-rabbit IgG (Invitrogen). Sections were examined with an LSM510 or LSM610 confocal microscope (Carl Zeiss).

### Measurement of infarction volume

Three 30-μm coronal sections at the levels of 1.7 mm, 0.7 mm, −0.3 mm, −1.3 mm, −2.3 mm, and −3.3 mm from bregma were cut and mounted on glass slides (Marienfeld). The sections were stained with 0.2% cresyl violet (Sigma-Aldrich) and imaged using a dissecting microscope equipped with a digital camera (Olympus, Tokyo, Japan). The size of the infarct area was measured using ImageJ v1.4 software (National Institutes of Health, Bethesda, MD, USA). Reductions in brain volume were calculated as follows:

Brain volume reduction = (infarct size in the ipsilateral hemisphere)/(size of the intact contralateral hemisphere).

### Magnetic resonance imaging (MRI)

All MRI experiments were performed at the Korea Basic Science Institute in Ochang, Korea, using a 4.7-T animal MRI scanner (BioSpec 47/40; Bruker, Germany) with 72-mm rat brain surface coils for radiofrequency transmission and reception, respectively.

All axial rat brain images were obtained using a rapid acquisition with relaxation enhancement pulse sequence with the following parameters: repetition time = 5 s, effective echo time = 90 ms, slice thickness = 1 mm, field of view = 3 × 4 cm^2^, matrix size = 256 × 256, number of averages = 4, and acquisition time = 10 min 40 s.

### Statistical analysis

Behavioral scores were evaluated using a repeated-measures ANOVA. Differences in the size of damaged brain areas between control and UCB-PL-treated groups were assessed using Student's *t*-tests. Differences between control, UCB-PL-treated, and aged-PL-treated groups were assessed using a one-way ANOVA followed by Student-Newman-Keuls *post hoc* tests. Differences were considered statistically significant at *p* < 0.05.

## CONCLUSIONS

Overall, our study reveals, for the first time, the therapeutic efficacy of UCB-PL in a rat model of ischemic stroke. Furthermore, we have provided valuable information regarding the possible mechanisms underlying the efficacy of UCB-PL. Because of the therapeutic potential and inherent advantages of UCB-PL, this therapy is a promising option for the treatment of acute ischemic stroke patients who are not eligible for intravenous thrombolytic therapy.
